# mGluR-Dependent Synaptic Plasticity in Drug-Seeking

**DOI:** 10.3389/fphar.2012.00159

**Published:** 2012-08-27

**Authors:** Camilla Bellone, Manuel Mameli

**Affiliations:** ^1^Department of Basic Neuroscience, University of GenevaGeneva, Switzerland; ^2^Institut du Fer à MoulinParis, France; ^3^INSERM, UMR-S 839Paris, France; ^4^Université Pierre et Marie CurieParis, France

**Keywords:** mGluR, synaptic plasticity, addiction, long-term depression, AMPA receptors, NMDA receptor

## Abstract

A primary feature of drug addiction is the compulsive use despite negative consequences. A general consensus is emerging on the capacity of addictive substances to co-opt synaptic transmission and synaptic plasticity in brain circuits which are involved in reinforcement and reward processing. A current hypothesis is that drug-driven neuroadaptations during learning and memory processes divert the functions of these brain circuits, eventually leading to addictive behaviors. Metabotropic glutamate receptors (mGluRs) not only lead to long-term modulation of synaptic transmission but they have been implicated in drug-evoked synaptic plasticity and drug-seeking behaviors in two important ways. mGluR-dependent modulation of synaptic transmission is impaired by drug experience but interestingly their activation has been indicated as a strategy to restore baseline transmission after drug-evoked synaptic plasticity. Here we focus on the cellular mechanisms underlying mGluR-dependent long-term changes of excitatory synapses, and review results implicating these receptors in drug-evoked synaptic plasticity.

## Introduction

Significant evidence is converging to the idea that glutamatergic transmission plays a pivotal role in addictive-like behaviors (Kalivas, 2004; Gass and Olive, 2008). Studies employing rodent models suggest the use of metabotropic glutamate receptor (mGluR) modulators as a therapeutic strategy in treating drug addiction (Spooren et al., 2003). Importantly, the potential use of mGluR ligands lies in understanding the function of mGluRs at synapses in the central nervous system. These receptors are G-protein-coupled and classified in three groups according to their sequence homology (Pin and Duvoisin, 1995). However, drug development and fundamental research have focused their attention on Group I (mGluR-I) and Group II mGluRs (mGluR-II) as potential therapeutic targets. For this reason, in the present review we will focus on the properties of mGluRs and on the implication of mGluR-I- and mGluR-II-dependent synaptic plasticity in the context of drug-seeking.

## Group I and II mGluRs

mGluR-I are postsynaptically enriched at glutamatergic synapses. They couple to G-proteins of the G_q_/G_11_ family and activate phospholipase Cβ leading to the hydrolysis of phosphotinositides followed by the generation of inositol 1,4,5-trisphosphate and diacylglycerol. This canonical signaling cascade triggers calcium mobilization and activation of a broad range of downstream effectors, including protein kinase C (PKC) pathway, the mitogen-activated protein kinase/extracellular receptor kinase pathway, and the mammalian target of rapamycin/p70 S6 kinase pathway (Hou and Klann, 2004; Page et al., 2006). Activation of these secondary signaling cascades by mGluR-I modulates both ion channel function and endocannabinoid (eCB) synthesis (Kammermeier et al., 2000; Galante and Diana, 2004).

mGluRs-I comprises the mGluR1 and mGluR5 which are broadly expressed in the brain. Subcellular electron microscopy localized these receptors on the postsynaptic membrane in the perisynaptic zone (Lujan et al., 1997) where they regulate both inhibitory and excitatory synaptic transmission. Although both receptors are G_q_-coupled, their signaling proprieties are different, suggesting distinct neuronal function (Choi et al., 2011).

mGluR-II (mGluR2 and mGluR3), are coupled to G_i/o_ proteins that inhibit adenylate cyclase and prevent the formation of cyclic adenosine 3′5′-monophosphate (cAMP). These receptors are predominantly expressed presynaptically (Niswender and Conn, 2010) and their activation can decrease neurotransmitter release via a cascade of events that engage voltage-gated calcium channels as well as SNAP-25-dependent mechanisms (Anwyl, 1999; Cartmell and Schoepp, 2000, Robbe et al., 2002a; Upreti et al., 2012).

The activation of these two subfamilies of mGluRs at excitatory synapses modulates the strength of synaptic transmission by inducing both long-term potentiation (LTP) and long-term depression (LTD) in various brain regions (Lüscher and Huber, 2010). In particular mGluR-dependent LTD has been extensively studied at various synapses and multiple induction mechanisms have been described to modulate the synaptic transmission in normal function as well as in diseases (Bellone et al., 2008).

## Synaptic Plasticity and Drug-Seeking

Addictive drugs target the mesocorticolimbic dopamine (DA) circuit (Lüscher and Ungless, 2006) that originates in the ventral tegmental area (VTA), where DA neurons project mainly to the nucleus accumbens (NAc) and prefrontal cortex (PFC). A common feature of addictive drugs is their ability to trigger an increase in extracellular DA levels in VTA and projecting areas, and to cause adaptations both at glutamatergic and GABAergic synapses (Kauer and Malenka, 2007). These drug-evoked synaptic adaptations are, in the long run, able to reorganize the neural circuits, and may represent a fundamental cellular mechanism leading to addictive behavior (Lüscher and Malenka, 2011).

In the specific case of cocaine, it has been shown that acute and chronic drug exposure increases the ratio of AMPA and NMDA receptor mediated currents in different brain areas, suggesting an increase in synaptic efficacy at excitatory inputs (Thomas et al., 2001; Ungless et al., 2001, Dumont et al., 2005; Bellone and Lüscher, 2006).

**Figure 1:**
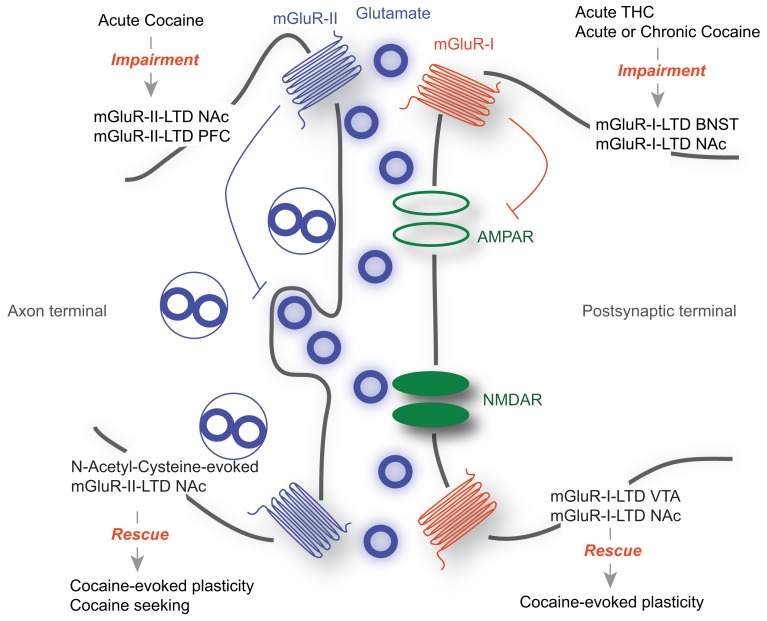
**The role of mGluR-LTD during drug-seeking**. mGluR-I are mainly located at the postsynaptic membrane, while mGluR-II are mostly presynaptic. While mGluR-I-LTD has been extensively described as postsynaptic and occurring through the removal of AMPARs, mGluR-II LTD expression is presynaptic, involving a decrease in neurotransmitter release. Acute drug exposure or withdrawal abolishes mGluR-LTD in various structures including the BNST and the NAc (Top). On the other hand forms of mGluR-LTD are able to rescue drug-evoked synaptic plasticity and behavioral adaptations (Bottom).

Along with changes in AMPA and NMDA receptor mediated transmission, drug exposure alters mGluR-dependent synaptic plasticity. mGluRs-I and mGluRs-II have been implicated in these phenomena, as well as in drug-evoked behavioral adaptations (Olive, 2009) (Figure 1). However, a causal link between the synaptic and the behavioral observations remains elusive.

## Drug Exposure Impairs mGluR-Dependent Plasticity

Addictive drugs alter mGluR-dependent synaptic plasticity in brain regions related to the mesolimbic system. In the NAc, the pharmacological activation of mGluRs-I by the agonist (S)-3,5-dihydroxyphenylglycine (DHPG) promotes release of eCBs and reduces glutamate release via the activation of cannabinoid 1 (CB1) receptors, leading to a presynaptic form of LTD (Fourgeaud et al., 2004). A single *in vivo* exposure to cocaine or delta-tetrahydrocannabinol transiently impairs this form of mGluR-LTD (Fourgeaud et al., 2004; Mato et al., 2004) via the reduction of mGluR5 expression and the dysfunction of CB1Rs (Fourgeaud et al., 2004; Mato et al., 2004). The intermediate role of D1 receptor signaling seems to play a fundamental role in this cascade. The abolishment of this form of plasticity in the NAc may represent a mechanism to counteract the decrease in glutamatergic activity observed after cocaine exposure (Pierce et al., 1996). However, the overall cellular mechanism underlying cocaine-evoked reduction of mGluR5 protein and signaling remains unclear. Importantly, interplay between mGluRs and eCB may exist. As recently shown, a single cocaine injection impairs eCB-LTD in D2-containing MSNs in the NAc suggesting that cocaine may disrupt eCB-dependent synaptic plasticity via alterations in mGluR-signaling (Grueter et al., 2012).

An important role of mGluR5 has been as well established in the bed nucleus of stria terminalis (BNST), an integrative center implicated in stress and rewarding responses (Jalabert et al., 2009). While a single cocaine injection does not alter mGluR-I-LTD in this region, chronic drug treatment impairs this form of plasticity 24 h after the last injection (Grueter et al., 2006). Indeed, decreasing the function *in vivo* of mGluR5 by using the specific antagonist MPEP, prevented cocaine-induced abolishment of the mGluR-I-LTD (Grueter et al., 2008). Although the cascade of events has been partially characterized, it is so far unclear whether cocaine prevents the mGluR-I-LTD or whether it engages mGluR-dependent signaling occluding the synaptic plasticity. Therefore the mechanisms by which drug experience impairs mGluR-I-LTD in the BNST remain in part elusive.

The cellular adaptations described above occur days after drug exposure in virtually all treated animals and therefore they could represent an early mechanism for priming addictive behaviors. A critical step in the development of the disease is the shift from a recreational and controlled drug use to a compulsive drug taking despite negative consequences (American Psychiatric Association, 1994). Addiction can be modeled in animals, and rodent models have been established reflecting the human conditions. Indeed, only a restricted number of animals fulfill the hallmarks of drug addiction (“addict” vs. “non-addict”) suggested by the Diagnostic and Statistical Manual of Mental Disorders (Deroche-Gamonet et al., 2004). This animal model has been recently employed to identify the biological traits of individual vulnerability to addiction.

Excitatory synapses onto MSN neurons of the NAc undergo two different forms of LTD: one is induced by mGluR-II activation and expressed presynaptically while the other is induced by NMDAR activation and expressed postsynaptically. While in “non-addict” and “addict” animals the mGluR-II-LTD is unaffected, NMDAR-LTD is abolished in “addict” rats (Kasanetz et al., 2010). Interestingly, rats with a short history of cocaine seeking showed impaired NMDA-LTD, suggesting that while in “non-addict” rats synaptic function goes back to baseline, in “addict” rats the NMDA-LTD remains impaired. This suggests that this “anaplasticity,” the inability to rescue normal baseline synaptic transmission, may represent a cellular locus for the shift to compulsive drug use (Kasanetz et al., 2010).

The glutamatergic afferents so far described remain elusive, however there are reasons to believe that they might be arising from cortical brain areas. Indeed, modulation of cortical inputs onto MSNs rescues cocaine-evoked synaptic and behavioral adaptations (Pascoli et al., 2012). Furthermore cortical brain areas are important in the control of drug intake (Kalivas et al., 2005). This evidence raises the hypothesis that alteration in mGluR-dependent synaptic plasticity can as well occur within the PFC. In a recent study, cocaine self-administration, in both “addict” and “non-addict” rats abolished the eCB-LTD in the PFC (Lafourcade et al., 2007). On the other hand, a decrease in mGluR2/3 protein levels in “addict” animals impaired the induction of mGluR-II-LTD (Kasanetz et al., 2012). The specific impairment of mGluR-II-LTD in this animal model provides important insight for the role of mGluRs during the development of addiction. The observation in NAc and PFC supports a scenario in which the impairment of long-term synaptic plasticity in “addict” animals may represent a substrate for transition to addiction in interconnected structures of the mesocorticolimbic circuit.

As described above, many evidences described the impairment of mGluR-LTD in various regions of the mesocorticolimbic system at different time points of drug use. The interpretation of the data is however not trivial. How mGluR-LTD impairment may account for the development of complex behavior such as compulsive drug use? Linking “anaplasticity” with lack of flexibility in controlling drug use over time, while intriguing, may not represent the only explanation. Future studies would need to assess the cellular, molecular, genetic adaptations that occur in parallel with the impairment of mGluR-LTD during the development of drug addiction.

## Rescue of Cocaine-Evoked Plasticity by mGluRs

While many different forms of drug-evoked synaptic plasticity have been described in the mesolimbic system, several observations point to the capacity of mGluRs to reverse cocaine-evoked adaptations both in the VTA and in the NAc.

Drug-evoked synaptic plasticity occurs in the VTA within a few hours after drug exposure, it persists for about a week, and it is permissive for further adaptations in the NAc, suggesting a hierarchy in the drug-evoked synaptic plasticity (Argilli et al., 2008; Mameli et al., 2009). Three different parameters of synaptic transmission change early on after drug exposure. First, a single injection with cocaine, morphine, ethanol or nicotine increases the AMPA to NMDA ratio at excitatory synapses onto DA neurons (Ungless et al., 2001; Saal et al., 2003). This occurs concomitantly with a change in the subunit composition of AMPAR-mediated responses (Bellone and Lüscher, 2006). Indeed, the current-voltage relationship of AMPA receptor responses shows an inward rectification suggesting the insertion of GluA2-lacking calcium permeable AMPA receptors (CP-AMPARs) after drug exposure. The use of two-photon laser photolysis of caged glutamate further identified a decrease in function of NMDARs and altogether these synaptic changes contribute to invert learning rules (Mameli et al., 2011).

Interestingly the CP-AMPARs can be removed from the synapses both *in vitro* and *in vivo*. In slices from mice treated with a single cocaine injection, the agonist of mGluR-I, DHPG, induces a form of LTD that is expressed by the removal of CP-AMPARs and the insertion of calcium impermeable GluA2-containing (CI)-AMPARs (Bellone and Lüscher, 2006). This suggests that drug exposure and in turn the presence of CP-AMPARs reveals a previously absent mGluR-LTD at these synapses. The mGluR-I-driven switch in AMPAR subunit composition restores baseline transmission and it relies on the *de novo* synthesis of GluA2 protein (Mameli et al., 2007). Since NMDAR function also changes after drug exposure, it will be important in the future to investigate whether the activation of mGluR-I also rescues transmission of NMDARs. Reversal of cocaine-induced synaptic plasticity occurs *in vivo* as well. Injection of a positive allosteric modulator of mGluR1 is able to remove CP-AMPARs, restoring basal transmission, and preventing more widespread changes downstream to the NAc (Bellone and Lüscher, 2006; Mameli et al., 2009). These observations led to the hypothesis that mGluR-I modulation represents a pharmacological tool to revert early drug-evoked plastic changes. Three major questions arise from these studies: (i) how does cocaine alter mGluR-signaling pathway? (ii) does the mGluR-I-LTD after insertion of CP-AMPARs occurs in other brain structures? and (iii) what is the behavioral implication of these early synaptic adaptations and its rescue by mGluR-I activation?

Susceptibility to relapse is a core component in the addiction process. Relapse can be modeled in rodents by exposing animals to drug-related cues after long withdrawal periods following self-administration, a paradigm termed incubation of craving (Lu et al., 2004). In acute slices containing NAc, synaptic GluA1 protein increases after 45 days of withdrawal following cocaine self-administration. These receptors were functional at synapses as indicated by the presence of inwardly rectifying AMPA-mediated currents and by the sensitivity to specific CP-AMPARs open-channel blockers (Conrad et al., 2008; Mameli et al., 2009, McCutcheon et al., 2011b). In conclusion, the switch from CI-AMPARs to CP-AMPARs at excitatory synapses onto MSN neurons of the NAc, may represent a cellular substrate for cocaine seeking. In addition to these observations, it has been recently shown that a new form of mGluR-I-LTD occurs after cue-induced cocaine craving in the NAc. While the activation of mGluR5 no longer induces the eCB-mediated LTD that is generally observed in drug naïve animals, mGluR1 activation induces a form of LTD that reverses the cocaine-induced accumulation of CP-AMPARs, restoring the initial AMPA-mediated transmission (McCutcheon et al., 2011a). This form of plasticity in the NAc depends on calcium/PKC signaling and therefore shows many common features with the mGluR-LTD described in the VTA (Bellone and Lüscher, 2005). Two important points remain still elusive: how does mGluR-signaling change upon cocaine experience? Is the mGluR-LTD a general feature of D1- and D2-expressing neurons in the NAc? These results together with future studies may provide insights to the appealing hypothesis that mGluR1s represent a gate controlling the levels of CP-AMPARs vs. CI-AMPARs. Indeed, a single drug exposure only transiently drives CP-AMPAR insertion in DA neurons of the VTA. In this case mGluR tone allows the system to recover baseline transmission in about a week. However, chronic cocaine treatment or self-administration overrides this key function such that endogenous activation of mGluR1 is not enough to restore basal transmission. This mediates more persistent changes in the VTA and the occurrence of synaptic adaptations in the NAc. Pharmacological boosting of mGluR1 function enables mGluR-I-LTD and in turns rescues baseline synaptic transmission in both VTA and NAc, suggesting a potential therapeutic impact at the behavioral level.

A further important role of mGluR-I-mediated plasticity has been recently shown at the developmental stage. The postnatal development of glutamatergic transmission requires mGluR1 activation in the VTA switching CP-AMPARs to CI-AMPARs (Bellone et al., 2011). *In utero* cocaine exposure delays the synaptic maturation of both AMPAR- and NMDAR-mediated transmission in the offspring such that excitatory synapses onto DA neurons show CP-AMPARs and GluN2B containing NMDARs beyond P14. Interestingly, the activation of mGluR1 *in vivo* by an allosteric modulator rescues the synaptic impairment induced by cocaine by inserting CI-AMPARs and GluN2A NMDARs. These observations led to the conclusion that in offspring from cocaine-exposed mothers, mGluR1 becomes inefficient to drive the postnatal maturation.

The role of rescuing drug-evoked synaptic abnormalities has also been proposed for mGluR-II. Repeated cocaine exposure alters the function of mGlu2/3 receptors and impairs the mGluR2/3-dependent plasticity both in the PFC and NAc (Xi et al., 2002; Huang et al., 2007, Xie and Steketee, 2008). As a consequence, both LTP and LTD are impaired in rats extinguished from cocaine self-administration for at least 3 weeks (Moussawi et al., 2009). Therefore, restoring the tone of mGluR2/3 would be able to rescue the ability of the excitatory synapses to induce LTP as well drug-seeking behaviors. For this purpose, *N*-acetylcysteine has been used as an indirect agonist of mGluR2/3 to restore glutamate homeostasis (Baker et al., 2003; Zhou and Kalivas, 2008). Acute administration of *N*-acetylcysteine has been shown to prevent drug-seeking and to reverse synaptic transmission in NAc. Interestingly the activation of different mGluRs has opposite effects; while the direct pharmacological activation of mGluR2/3 prevents the relapse in the reinstatement model of cocaine, the activation of mGluR5 does not inhibit the cocaine seeking behaviors. On the contrary, block of mGluR5 prevents cocaine reinstatement for drug-seeking, suggesting dissociation between synaptic plasticity, and behavioral models. These somehow contrasting data require a further investment in the understanding of the roles of different mGluR subfamilies in modulating of drug-seeking behaviors.

## Concluding Remarks

During the past decade our understanding of mGluR function has led to the development of a wide range of pharmacological and genetic tools that contributed to the discovery of the role of mGluRs in brain function. Many behavioral evidences suggested an important role of mGluR-signaling in cocaine reinforcement as well as cocaine extinction and reinstatement (Kumaresan et al., 2009; Wang et al., 2012). We focused here on mGluR-dependent plasticity, a key player in learning processes as well as in disorders of the nervous system. Much experimental evidence highlights the role of mGluR-LTD in mediating early (acute drug exposure) and late processes (chronic and self-administration paradigms), during the etiology of addictive behaviors. A big challenge in the field is to directly link specific synaptic modification to specific behaviors and, while a large effort has focused on the drug-evoked changes in mGluR-LTD after acute exposure to addictive substances, much work is still required to better probe the importance of mGluR-LTD during the late phase of the disease. Future studies will need to investigate how changes in synaptic plasticity can lead to drug addiction. A feature in the etiology of addictive behaviors is the recruitment in a hierarchical fashion from ventral (VTA, substantia nigra, NAc) to dorsal (dorsal striatum) brain structures (Belin and Everitt, 2008; Mameli et al., 2009). mGluRs are largely expressed in the striatum, a region implicated in goal-directed behaviors, however the links between mGluR-dependent plasticity in this region and drug-seeking remain yet unknown.

Another important component of mGluR-I activation in the midbrain and in the striatum is the synthesis of eCB, which mediate often short- as well as long-term presynaptic forms of plasticity. Drug exposure generally leads to an impairment eCB-LTD at both inhibitory and excitatory synapses, an effect that may contribute to drug-evoked behavioral adaptations. Further characterization of the interactions mGluRs, eCB, and synaptic plasticity in the context of drug-seeking would be certainly important to probe the potential benefits of compounds targeting CBRs and eCB signaling.

Finally determining the behavioral consequences of mGluR-LTD-dependent rescue of plasticity and understanding whether inter-individual vulnerability to addiction lies in genetic, environmental-linked variation in mGluR function represents the major breakthroughs for the development of therapeutic strategies of addictive behaviors.

## Conflict of Interest Statement

The authors declare that the research was conducted in the absence of any commercial or financial relationships that could be construed as a potential conflict of interest.
